# Supplementary material for: Could an Internet-Based Foot–Ankle Therapeutic Exercise Program Modify Clinical Outcomes and Gait Biomechanics in People with Diabetic Neuropathy? A Clinical Proof-of-Concept Study
Source: Sensors (Basel). 2022 Dec 7;22(24):9582. doi: 10.3390/s22249582 (PMC9781221; doi:10.3390/s22249582)
Supplement: Supplementary file 1 [file sensors-22-09582-s001.zip › sensors-2014811-supplementary.pdf]

Be aware of the appearance of injuries on your foot.

Failure to control hyperglycemia can compromise blood circulation and nerves, leading to a loss of sensitivity. Usually, in these patients, the injury begins with the appearance of calluses, cracks, blisters, chilblains, scratches, cuts, or foreign bodies that put the person at risk of developing an ulcer.

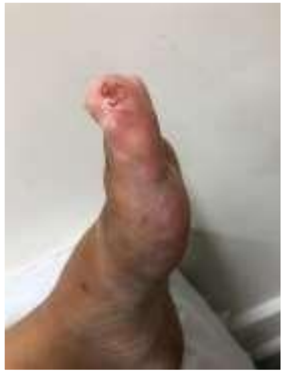

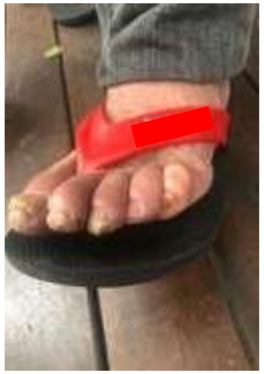

Source: personal archive.

What to do after the appearance of an ulcer?

In the event of any foot injury (blisters, calluses, cracks and cuts) and/or the presence of an open wound (ulcer) inform us immediately and look for the health unit closest to your residence.

E-mail:focarct@gmail.com

Researchers: Érica Queiroz (11) 98549-2016

Jane Ferreira (11) 9.8287 9811

Department of Physiotherapy, Speech and Occupational Therapy
School of Medicine
University of Sao Paulo

Research project:

"Rehabilitation technologies for preventing impairments in people with diabetes: FootCAre (FOCA) randomized preventive trials"

Rua Cipotânea, nº 51 – Cidade Universitária
Cep.05360-160 – São Paulo – SP
Tel.(11) 3091-8426 – Fax. (11) 3091-7462
E-mail: labimphfofito@gmail.com

Foot care[1]

1. Regularly inspect your feet for bruises or cuts. Also look for anything inside your shoes that could injure your foot.

If necessary, use a mirror to check the soles. of the feet.

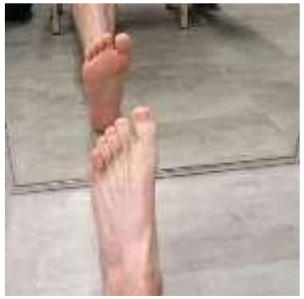

Source: personal archive.

2. Be aware of any changes in your fingers and feet, such as: deformities; calluses; bubbles; ingrown toenails; dry skin and cracks, as these changes can cause wounds/ulcers.

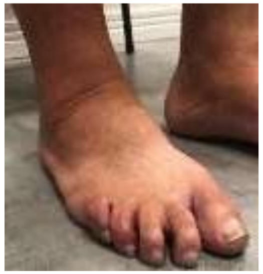

Source: personal archive.

3. To protect your feet, avoid walking barefoot, wearing socks or thin-soled shoes.

4. When washing your feet, dry your feet thoroughly and even between your toes.

5. Use moisturizer when the skin is dry to prevent cracking.

6. Cut fingernails properly (straight cut) to avoid ingrown nails.

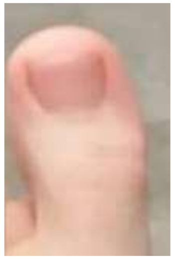

Source: personal archive.

7. Monitor whether the foot skin temperature is warm. The goal is to identify the early signs of inflammation.

8. If you have a wound/ulcer that is not healing, seek medical attention quickly.

In cases of any changes in your feet, seek help from a health professional.

[1] Bus SA, van Netten JJ, Lavery LA, Monteiro-Soares M, Rasmussen A, Jubiz Y, Price PE; International Working Group on the Diabetic Foot. Diabetes Metab Res Rev. 2016 Jan;32 Suppl 1:16-24.
